# Breakdown of long-range spatial correlations of infraslow amplitude fluctuations of EEG oscillations in patients with current and past major depressive disorder

**DOI:** 10.3389/fpsyt.2023.1132996

**Published:** 2023-04-26

**Authors:** Duho Sihn, Ji Sun Kim, Oh-Sang Kwon, Sung-Phil Kim

**Affiliations:** ^1^Department of Biomedical Engineering, Ulsan National Institute of Science and Technology, Ulsan, Republic of Korea; ^2^Department of Psychiatry, College of Medicine, Soonchunhyang University Cheonan Hospital, Cheonan, Republic of Korea

**Keywords:** spatial correlation, EEG amplitude fluctuation, depression, infraslow activity, theta oscillation, alpha oscillation

## Abstract

**Introduction:**

Identifying biomarkers for depression from brain activity is important for the diagnosis and treatment of depression disorders. We investigated spatial correlations of the amplitude fluctuations of electroencephalography (EEG) oscillations as a potential biomarker of depression. The amplitude fluctuations of EEG oscillations intrinsically reveal both temporal and spatial correlations, indicating rapid and functional organization of the brain networks. Amid these correlations, long-range temporal correlations are reportedly impaired in patients with depression, exhibiting amplitude fluctuations closer to a random process. Based on this occurrence, we hypothesized that the spatial correlations of amplitude fluctuations would also be altered by depression.

**Methods:**

In the present study, we extracted the amplitude fluctuations of EEG oscillations by filtering them through infraslow frequency band (0.05–0.1 Hz).

**Results:**

We found that the amplitude fluctuations of theta oscillations during eye-closed rest depicted lower levels of spatial correlation in patients with major depressive disorder (MDD) compared to control individuals. This breakdown of spatial correlations was most prominent in the left fronto - temporal network, specifically in patients with current MDD rather than in those with past MDD. We also found that the amplitude fluctuations of alpha oscillations during eye-open rest exhibited lower levels of spatial correlation in patients with past MDD compared to control individuals or patients with current MDD.

**Discussion:**

Our results suggest that breakdown of long-range spatial correlations may offer a biomarker for the diagnosis of depression (current MDD), as well as the tracking of the recovery from depression (past MDD).

## 1. Introduction

Finding biomarkers for depression from brain activity is important for the diagnosis of depression (1). Many biomarkers for depression using electroencephalogram (EEG) have been identified since several decades ago. A major biomarker for Major Depressive Disorder (MDD) in the current field is frontal alpha asymmetry, in which the magnitudes of alpha oscillations in the left and right hemispheres are more asymmetrical in patients with depression than in healthy individuals (2–5). This biomarker is known to be effective not only for diagnosis but also for treatment: Neurofeedback training to self-modulate the magnitudes of alpha oscillations in the left and right hemispheres to achieve better symmetry has been found to be effective in treating depression (6, 7), while not affecting mood in healthy participants (8). However, there has been inconsistencies in studies of frontal alpha asymmetry (5, 9); left prefrontal alpha activity was less than right prefrontal alpha activity in some studies, but could not be reproduced in other studies. Therefore, it is desirable to identify other EEG-based biomarkers that can be referenced to.

**Figure 1:**
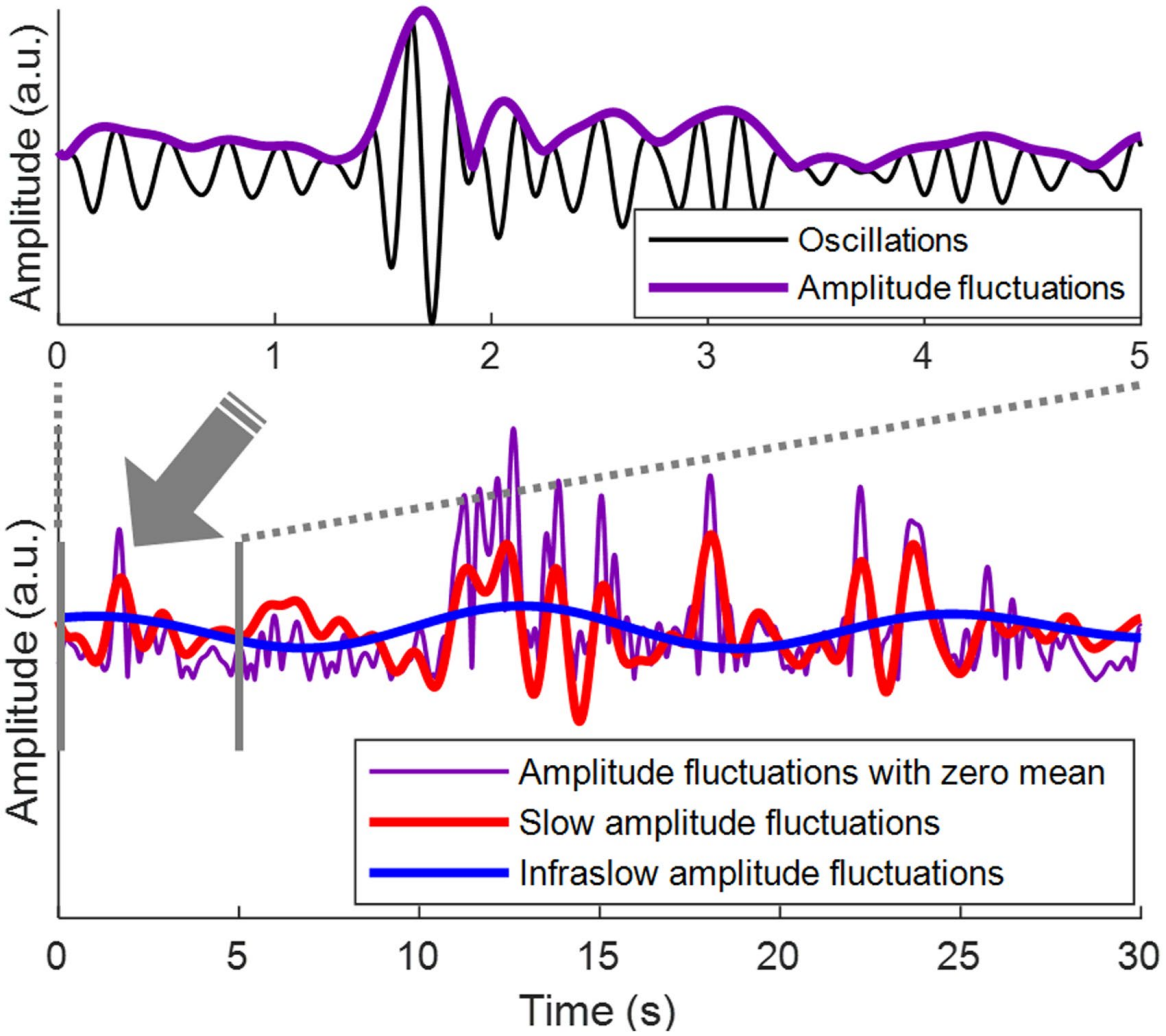
Amplitude fluctuations of EEG oscillations. (Top) Theta oscillations (3–7 Hz) are shown in black. Amplitude fluctuations of the theta oscillations (= the envelope of the theta oscillations) are shown in purple. (Bottom) The top panel is the first 5 s from the bottom panel. Amplitude fluctuations with zero mean are shown in purple. Slow amplitude fluctuations are obtained by bandpass filtering of amplitude fluctuations at 0.1–1 Hz, shown in red. Infraslow amplitude fluctuations are obtained by bandpass filtering of amplitude fluctuations at 0.05–0.1 Hz, shown in blue.

Here, we investigated whether spatial correlations of amplitude fluctuations of EEG oscillations could be a novel biomarker for depression. Amplitude fluctuations refer to fluctuations in the envelope of relatively fast EEG oscillations (>1 Hz) (Figure 1, top). During rest, these amplitude fluctuations mainly display infraslow frequencies (<0.1 Hz) (10). In this infraslow frequency range, amplitude fluctuations over a wide range of brain areas are correlated with each other, establishing long-range spatial correlations of amplitude fluctuations (11–13).

Correlations of amplitude fluctuations during rest have also been observed over long periods of time, namely, long-range temporal correlations, which represent correlations between amplitude fluctuations in different time periods at the same brain region (10). The temporal correlations have often been shown to indicate rapid reorganization of the brain networks (10, 14). In patients with MDD, long-range temporal correlation of amplitude fluctuations during rest is reportedly disrupted (15). In general, changes in structural connectivity in the brain are likely to induce the disruption of long-range temporal correlations (16), which suggests that MDD may alter structural connectivity (17). The disruption of long-range temporal correlations indicate that the amplitude fluctuations of EEG oscillations would become close to a random process. Accordingly, the spatial correlations of amplitude fluctuations over a wide range of brain regions, which indicate functional organization of the brain networks (11, 12), can also be affected by changes in structural connectivity (18). In this study, we hypothesize that not only long-range temporal correlation but also wide-range spatial correlation of amplitude fluctuations would be broken during rest in patients with MDD. The current study focused on the infraslow frequency band of EEG amplitude fluctuations. Our focus on the infraslow frequency band is motivated by previous findings that the infraslow frequency band of EEG amplitude fluctuations form the spatial correlation among brain regions in healthy subjects (11–13). As such, we aim to examine whether such spatial correlations would be disrupted in patients with MDD. Similar breakdowns of spatial correlations can also occur in other frequency bands, but are beyond the scope of this study. On the one hand, it is unclear of which frequency band the amplitude fluctuations of EEG oscillations would exhibit this disruption. To address this, we investigate spatial correlations of amplitude fluctuations over multiple frequency bands, including theta, alpha, and beta oscillations, which have been examined in the previous study on disruption of long-range temporal correlation in patients with MDD (15). We expected that if we could identify the breakdown of long-range spatial correlations, we might obtain additional evidence about changes in structural connectivity in patients with MDD. It has been reported that changes in structural connectivity can lead to the disruption of long-range temporal correlations (16). Therefore, our result showing the disruption of long-range temporal correlations would support that structural connectivity of patients with MDD may have changed (17). Also, spatial correlations of amplitude fluctuations, which represent the functional organization of brain networks (11, 12), were altered in patients with MDD, indicating altered structural connectivity (18).

Classification of patients with MDD relative to healthy individuals using EEG has been well established using advanced machine learning algorithms (19–21). It reflects that automated diagnosis of MDD using EEG is highly plausible in clinical examinations (19, 20). On the other hand, it remains to be challenging to distinguish between patients with past MDD [individuals who are not currently suffering from MDD but have had a history of MDD in the past; (22)] and current MDD (patients currently suffering from MDD) based on depressive symptoms in the case that patients with past MDD still present with depressive symptoms. No study has addressed whether it is possible to distinguish between past and current MDD based on the analysis of EEG. Brain structure (23) as well as functional connectivity measured by EEG (24) have been found to remain altered in patients with past MDD. Therefore, we hypothesize that the way long-range spatial correlations of amplitude fluctuations are disrupted—in terms of brain regions and frequency bands—might be specific to each of the past and current MDD. One possibility is that disruption of spatial correlations in patients with past MDD occurs in the same frequency band of EEG oscillations and between the same brain regions as that in patients with current MDD, but the extent to which spatial correlations are disrupted is alleviated, being closer to healthy individuals. Another possibility is that progressive reorganization of structural and functional connectivity in patients with past MDD is manifested in spatial correlations of different EEG frequency bands and brain regions from those in patients with current MDD. The present study examines these possibilities to find differences between past and current MDD in the disruption of long-range spatial correlations, which potentially would provide a new biomarker to track the progress of the recovery from MDD.

## 2. Materials and methods

### 2.1. Dataset

We used a dataset of 121 participants containing electrophysiological data and Beck depression inventory (BDI) scores (25). This dataset is publicly available at https://openneuro.org/datasets/ds003478/versions/1.1.0. Participants performed the experimental task of opening and closing their eyes several times during rest. The average sum of lengths of the periods with eyes closed and opened were 91.82 ± 23.49 s and 93.68 ± 24.81 s, respectively. Electrophysiological data were collected while participants performed experimental tasks. The electrophysiological data contain 64-channel EEG (Synamps 2, Neuroscan, Australia) and 2-channel electrooculograms sampled at 500 Hz. The reference channel of EEG was located between the Cz and CPz channels. The average total length of the EEG recordings in each session across participants was 326.07 ± 155.86 s. Recording sessions were held twice for each participant.

### 2.2. Classification of individuals with depression

Beck et al. (26) reported that a BDI score (27) of less than 10 could be classified as a healthy individual. Lasa et al. (28) reported that a BDI score of 13 or higher could be classified as depression. We referred to these studies to classify 121 participants in the dataset used in this study. Because there was no participant with a BDI score between 7 and 10, we classified participants with a BDI score of 7 or less as the control group (Control, *N* = 75). Participants with a BDI score of 13 or higher were classified into the depressive symptoms group (Depressive, *N* = 46). 32 participants in the Depressive group underwent a clinical interview. Among the 32 participants, 11 participants met the criteria of current major depressive disorder (cMDD, *N* = 11), 12 participants met the criteria of past major depressive disorder (pMDD, *N* = 12) and 9 participants did not meet the criteria of either (None, *N* = 9). Specifically, the cMDD group consists of patients currently suffering from major depressive disorder. The pMDD group consists of individuals who are not currently suffering from major depressive disorder but have had a history of major depressive disorder in the past. The None group consists of individuals who have depressive symptoms but have not suffered from major depressive disorder either in now or the past.

### 2.3. EEG signal processing

Artifacts were observed through visual inspection of the EEG, located at the beginning or end of the EEG recording. We removed these artifacts by eliminating the initial and final 10-s periods of the EEG recording. Independent component analysis (29) was performed to remove eye blink artifacts in the EEG.

We reduced the volume conduction effect using the surface Laplacian method before computing spatial correlations. We performed the surface Laplacian method on the EEG data by using the CSD toolbox (30–32). The parameters of the CSD toolbox were set to *m* = 4 and *λ* = 10–5, following the previous EEG oscillation study (33). Among the 64 EEG channels, 4 channels (CB1, CB2, M1, M2) not included in the standard montage were excluded from the analysis to use the CSD toolbox. Therefore, we used 60 EEG channels for analysis.

In order to verify that true EEG signals were being collected, we performed frequency analysis using Fourier transform to examine whether the frequency components in EEG of the dataset used in this study were those typically observed in humans. We defined the typical frequency components according to the previous study (15), including theta, alpha, and beta oscillations. In a similar fashion to the previous study, the present study defined theta oscillations as 3–7 Hz, alpha oscillations as 8–12 Hz, and beta oscillations as 17–25 Hz. Then, we examined if the central frequencies of theta oscillation (i.e., 4–5 Hz) exhibited higher power than its peripheral frequencies (i.e., 3, 6–7 Hz), if the central frequencies of alpha oscillation (i.e., 9–11 Hz) exhibited higher power than its peripheral frequencies (i.e., 8, 12 Hz), and if the central frequencies of beta oscillations (i.e., 20–22 Hz) exhibited higher power than its peripheral frequencies (i.e., 17–19, 23–25 Hz), which has been regarded as typical frequency properties of human EEG. The frequency components were identified in terms of the magnitude of the Fourier transform of the EEG for each frequency. Fourier transform was performed using the fast Fourier transform function built in MATLAB.

Bandpass filtering was performed using a Hamming window-based finite impulse response filter (built in MATLAB, the function fir1.m and filtfilt.m [filtfilt(fir1(7 * fix(“sampling rate” / “first cut-off frequency”), [“two cut-off frequencies”] * 2 / “sampling rate”), 1, “data”)], MATLAB R2021b) to extract theta (3–7 Hz), alpha (8–12 Hz), and beta (17–25 Hz) oscillations. To detect amplitude fluctuations, the amplitude envelopes of theta, alpha and beta oscillations were calculated using Hilbert transform. Hilbert transform was performed using MATLAB (34). The amplitude envelope was defined as the absolute value of the Hilbert transformed complex number.

In order to verify whether the frequency components of the amplitude fluctuations are prominently observed in the infraslow frequency band as indicated in the existing literature (10), Fourier transform was used to identify the frequency components of the amplitude fluctuations. The frequency components were identified in terms of the magnitude of the Fourier transformed amplitude fluctuations for each frequency.

Bandpass filtering was performed using a finite impulse response filter to obtain infraslow (0.05–0.1 Hz) and slow (0.1–1 Hz) fluctuations of the amplitude envelopes, that is, infraslow amplitude fluctuations and slow amplitude fluctuations, respectively (Figure 1, bottom). To detect the oscillation phase of the amplitude fluctuations, Hilbert transform was applied to the infraslow and slow amplitude fluctuations. Since bandpass filtering was performed in the infraslow frequency band, the infraslow amplitude fluctuations after filtering had zero-mean (Figure 1). The amplitude fluctuations in Bottom of Figure 1 were also zero-meaned to directly compare them with zero-meaned infraslow amplitude fluctuations.

### 2.4. EEG amplitude fluctuation analysis

#### 2.4.1. Definition of spatial correlation between amplitude fluctuations

We estimated the spatial correlation between a pair of infraslow or slow amplitude fluctuations each from a different EEG channel, based on the oscillation phase difference measured at each time point. Rather than using Pearson’s linear correlation between the amplitude envelopes to measure the spatial correlation (11), the present study applied bandpass filtering (within infraslow or slow band) to the amplitude envelopes to extract oscillation phases. The difference between the oscillation phases at different spatial locations then represents the degree of spatial correlation [i.e., functional connectivity; (35, 36)], which can be measured at each time point. Spatial correlation can be measured as a correlation between two time series at two distinct locations (11). A high spatial correlation reflects that the amplitudes of the two signals increase or decrease together. For infraslow time series oscillating at the same frequency band, this high spatial correlation means that the oscillation phases of two bandpass filtered signals rise or fall together (37). Thus, the difference between the oscillation phases provides an estimate of instantaneous spatial correlation, useful for real-time processing. Note that we did not calculate a typical correlation measure for infraslow activity because we aimed to estimate instantaneous spatial correlation in real time, which can be helpful for future development of clinical monitoring systems.

Spatial correlation based on an oscillation phase difference at time t , $\psi \left(t\right)$ , between two phases $\theta_{1}\left(t\right)$ and $\theta_{2}\left(t\right)$ (radian) is defined as:

(1) $$\psi \left(t\right)=\frac{2\pi }{\left|\theta_{1}\left(t\right)-\theta_{2}\left(t\right)\right|+\pi }-1$$

Note that $\left|\theta_{1}\left(t\right)-\theta_{2}\left(t\right)\right|$ is symmetric with respect to $\theta_{1}$ and $\theta_{2}$ , and has the range of [0,π] . If a phase difference is at the minimum, i.e., $\left|\theta_{1}\left(t\right)-\theta_{2}\left(t\right)\right|=0$ , $\psi \left(t\right)=1$ . If a phase difference is at the maximum, i.e., $\left|\theta_{1}\left(t\right)-\theta_{2}\left(t\right)\right|=\pi$ , $\psi \left(t\right)=0$ . If two oscillations are correlated with zero time lag, their phase difference would be small, making $\psi \left(t\right)\;$ large. Thus, $\psi \left(t\right)$ represents the degree of the spatial correlation. After calculating $\psi \left(t\right)$ at each t , we calculated the median of $\psi \left(t\right)$ ( Mψ ) over the entire period of each task (i.e., eyes open and eyes closed):

(2) $$M\psi =\mathrm{median}\left\{\begin{array}{l} \psi \left(t\right)|t\in a\;particular\;\\ \mathrm{condition of the participant} \end{array}\right\}$$

Mψ was used as an estimate of a spatial correlation between a pair of EEG channels with a particular oscillation frequency (e.g., $\theta_{1}$ is a phase of theta oscillations in the F3 channel and $\theta_{2}$ is a phase of theta oscillations in the P4 channel during eyes closed) for each participant.

Using Mψ , we first confirmed that the spatial correlation between infraslow amplitude fluctuations was larger than that between slow amplitude fluctuations, as shown in the previous study (10). We compared Mψ of infraslow and slow amplitude fluctuations in the control group. The infraslow and slow mean Mψ s of every possible channel pair were estimated, for each combination of frequency band (i.e., theta, alpha, and beta) and task (i.e., eyes closed and open). In fact, previous studies have reported that spatial correlation appears mainly in the infraslow frequency band (11–13). Since we aimed to find how this reported spatial correlation changes, we were necessarily to focus on the infraslow amplitude fluctuations. In addition, we conducted a confirmative analysis to verify that infraslow amplitude fluctuations were larger than slow amplitude fluctuations. Taken together the confirmative analysis result and the results from previous studies, we focused all the subsequent analyses on infraslow amplitude fluctuations.

#### 2.4.2. Inter-regional exploration of spatial correlation breakdown

Next, to explore depression biomarker candidates, the disruption of spatial correlations in amplitude fluctuations of various frequency bands (theta, alpha, and beta) was investigated under the conditions with eyes-open and eyes-closed, respectively, where the disruption of spatial correlations was defined as significant decreases in Mψ of the experimental groups compared to the control group. For each combination of frequency band and condition (e.g., theta band with eyes-open), we calculated the Mψ for every possible channel pair in the region pair (8 regions, left frontal, right frontal, left temporal, right temporal, left parietal, right parietal, left occipital, and right occipital, which are commonly divided regions in the scalp EEG studies; _8_C_2_ = 28 region pairs in total) in every participant. Then, for each pair, we statistically evaluated a decrease in Mψ of each experimental group (three subgroups of the depressive symptom group: cMDD, pMDD, and None) compared to the control group. We counted the pairs showing significant decreases in Mψ in the experimental group and sought for a combination of frequency band and condition that exhibited the highest pair count.

To that end, all EEG channels were divided into 8 regions: (1) left frontal region: FP1, AF3, F7, F5, F8, F1, FC5, FC3, and FC1; (2) right frontal region: FP2, AF4, F2, F4, F6, F8, FC2, FC4, and FC6; (3) left temporal region: FT7, T7, and TP7; (4) right temporal region: FT8, T8, and TP8; (5) left parietal region: CP5, CP3, CP1, P7, P5, P3, and P1; (6) right parietal region: CP2, CP4, CP6, P2, P4, P6, and P8; (7) left occipital temporal region: PO7, PO5, PO3, and O1; and (8) right occipital temporal region: PO4, PO6, PO8, and O2. Among all possible pairs of regions (_8_C_2_ = 28 pairs), we identified the one that showed the highest significance.

**Figure 2:**
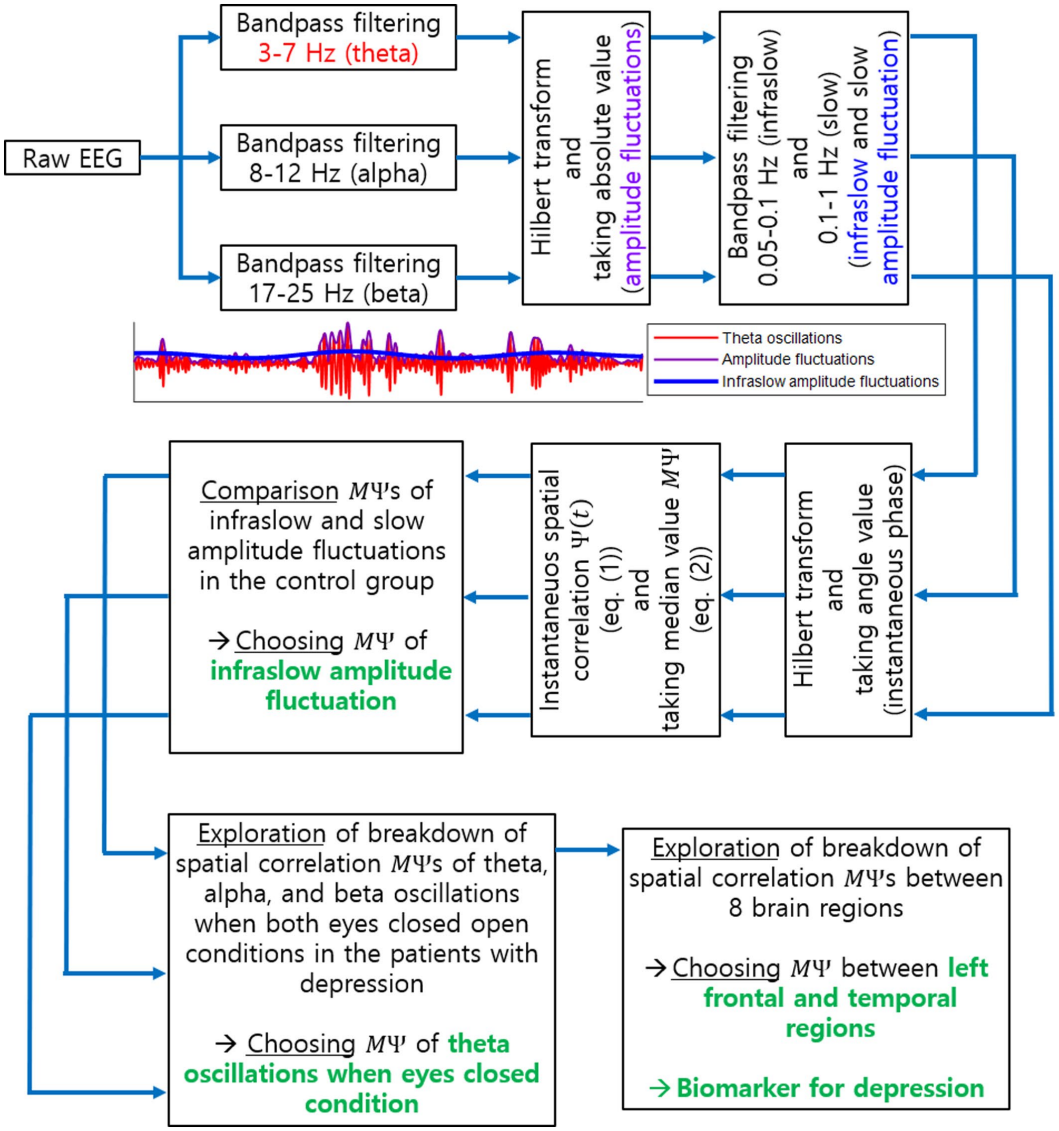
Procedure to explore a biomarker for current MDD using EEG spatial correlation. This illustrates all steps to identify the biomarker for current MDD as the breakdown of long-range spatial correlations. Specific EEG oscillation frequency band, task condition and brain regions, identified as a result of our investigation, are also illustrated in green.

In sum, our proposed biomarker is the disruption of spatial correlations between EEG channels in two different brain regions (among 8 regions above), where the disruption of spatial correlations was determined by significant decreases in Mψ of the infraslow amplitude fluctuation of EEG oscillations at a certain frequency band under a certain condition. Note that the evaluation of decreases in Mψ relies on comparison with the spatial correlation data in a healthy control group. The specific brain regions, frequency band, and condition for the proposed biomarker identified in this study will be described in the following section. An overall procedure to find the proposed biomarker is illustrated in Figure 2.

### 2.5. Discrimination of individuals using spatial correlations

The proposed biomarker was explored based on comparison between the experimental and control groups. To assess the feasibility of using this biomarker to diagnose an individual for cMDD or pMDD, we developed a simple procedure that discriminated an individual as either cMDD or not. Specifically, we discriminated each participant into cMDD or the other group (i.e., cMDD vs. pMDD, cMDD vs. None, and cMDD vs. Control) using Mψ data of the most significant channel pair in the all inter-regional pairs identified above. First, we employed a leave-one-subject-out scheme to assess our discrimination procedure using Mψ data. For each pair, we first calculated the median value of Mψ s over all subjects in each group, except for a test subject who was left out. Then, we determined which group the Mψ value of the test subject was closer to the median value of. The chance level of discrimination was 0.5. It is noteworthy that we designed a quite straightforward procedure to discriminate cMDD from other groups here, rather than employing more sophisticated classification algorithms, to evaluate a potential of the proposed biomarker to be directly used for diagnosis.

### 2.6. Statistical test

We did not assume a specific distribution of the population to perform statistical tests. Therefore, the one-tailed Wilcoxon rank-sum test was used for all comparisons, including the comparison between the infraslow and slow frequency bands in the control group, and the comparison between the control group and the experimental groups. To identify statistically significant channel pairs within each region pair, an FDR-corrected *p*-value of 0.05 was used.

## 3. Results

### 3.1. Frequency components of EEG and amplitude fluctuations during rest

**Figure 3:**
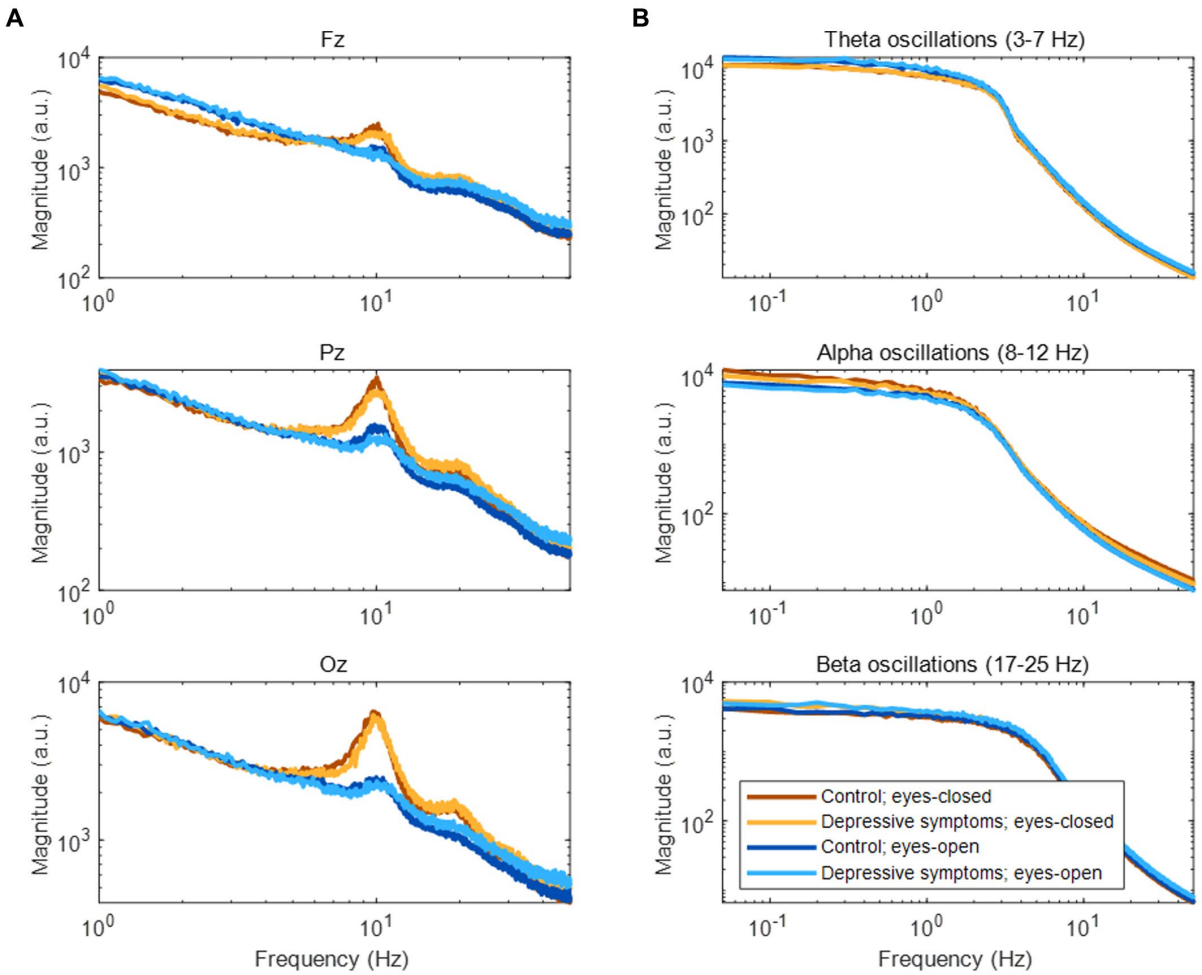
Frequency components of EEG and amplitude fluctuations of EEG oscillations. **(A)** Frequency components of EEG during rest. **(B)** Frequency components of amplitude fluctuations according to three EEG oscillations at the Fz channel.

In the frequency domain, the magnitude of EEG generally showed log–log linearity except for apparent peaks in the alpha (8–12 Hz) and beta (17–25 Hz) bands (See Figure 3A for representative examples). Although no clear peak was found in the theta band (3–7 Hz), we included it in our analysis based on previous studies (see Section 2.4). The amplitude fluctuations of theta, alpha, and beta oscillations mainly consisted of infraslow (0.05–0.1 Hz) and slow (0.1–1 Hz) frequency components (Figure 3B).

### 3.2. Disruption of spatial correlation of amplitude fluctuations in patients with major depressive disorder

We first tested which of the infraslow amplitude fluctuations and slow amplitude fluctuations had higher spatial correlation ( Mψ ) across each channel pairs (_60_C_2_ = 1770). In the control group, 1728/1770, 1763/1770, and 1749/1770 pairs were higher at the infraslow amplitude fluctuations than at the slow amplitude fluctuations in the theta, alpha, and beta oscillations in the eyes-closed conditions, respectively (rank-sum test, FDR-corrected *p* < 0.05). In the control group, 1393/1770, 1757/1770, and 1760/1770 pairs were higher at the infraslow amplitude fluctuations than at the slow amplitude fluctuations in the theta, alpha, and beta oscillations in the eyes-open conditions, respectively (rank-sum test, FDR-corrected *p* < 0.05). There was no significant channel pairs that the spatial correlation was higher at the slow amplitude fluctuations than at the infraslow amplitude fluctuations in all EEG oscillations in the all conditions (rank-sum test, FDR-corrected *p* < 0.05). In addition, previous studies also report that spatial correlation appears large in infraslow amplitude fluctuations (11–13). Therefore, we focused on the amplitude fluctuations at the infraslow frequency band. For slow amplitude fluctuations, see Supplementary material.

**Figure 4:**
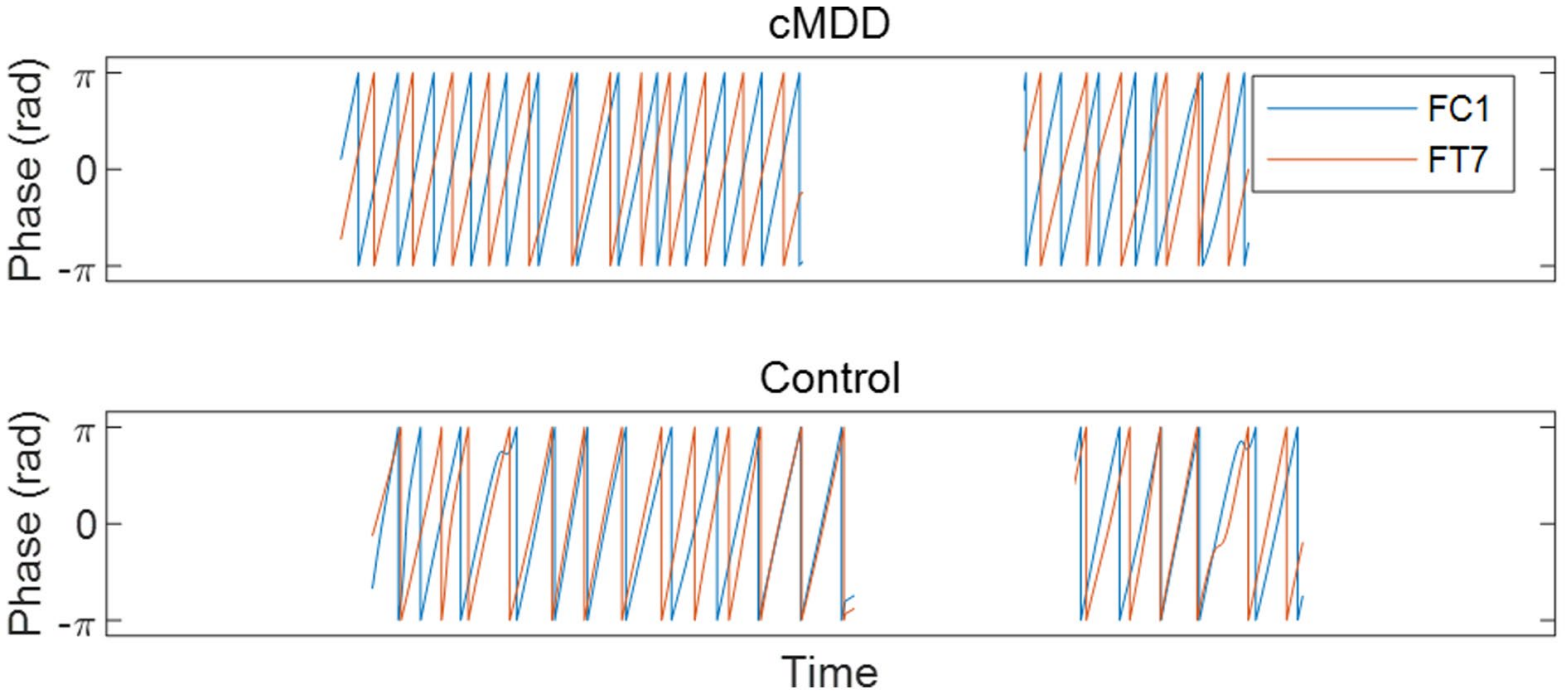
An example of the disruption of spatial correlation between amplitude fluctuations of theta oscillations in an eyes-closed patient with major depressive disorder. A graph is drawn only when the eyes are closed. Misaligned oscillation phases indicate the disruption of spatial correlation.

**Figure 5:**
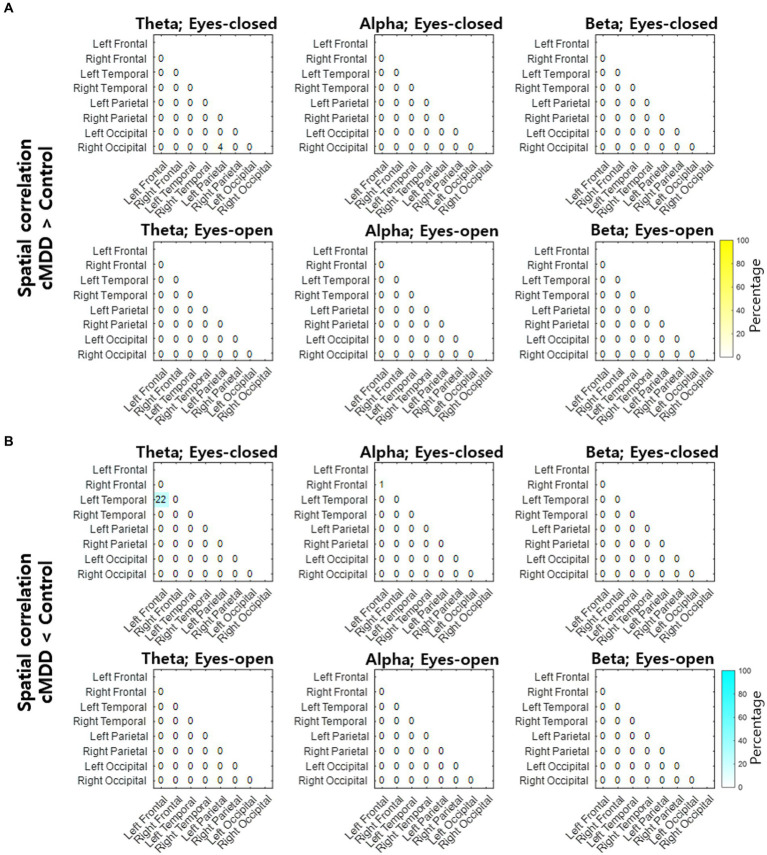
Differences of spatial correlation of infraslow amplitude fluctuations between current major depressive disorder and control groups. **(A)** The ratio (percentage) of channel pairs that the spatial correlation was significantly higher in cMDD than in control group (rank-sum test, FDR-corrected *p* < 0.05). **(B)** The ratio (percentage) of channel pairs that the spatial correlation was significantly lower in cMDD than in control group (rank-sum test, FDR-corrected *p* < 0.05).

**Figure 6:**
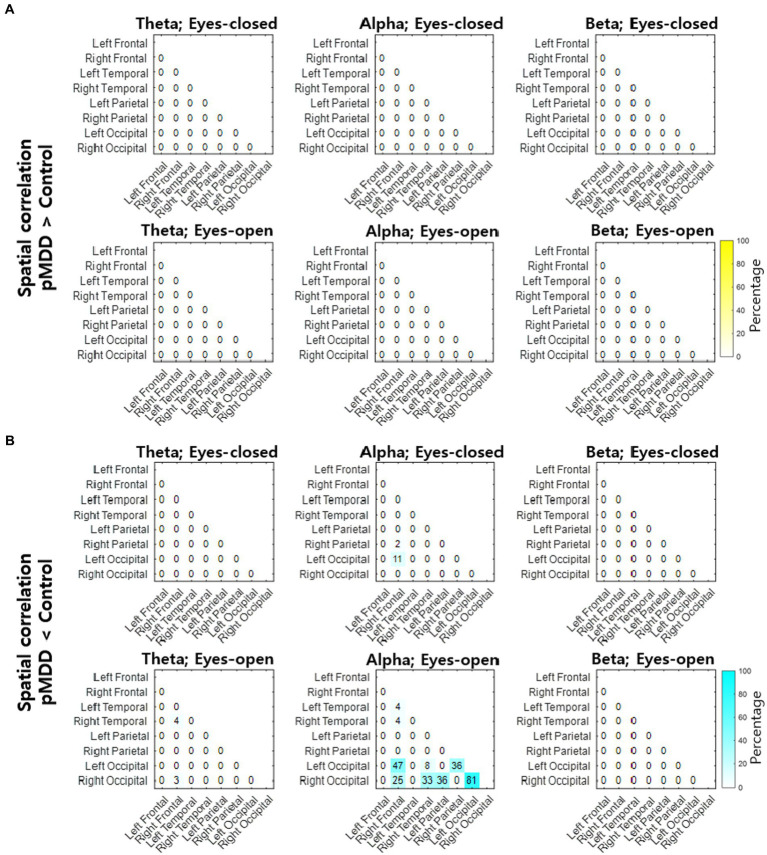
Differences of spatial correlation of infraslow amplitude fluctuations between past major depressive disorder and control groups. **(A)** The ratio (percentage) of channel pairs that the spatial correlation was significantly higher in pMDD than in control group (rank-sum test, FDR-corrected *p* < 0.05). **(B)** The ratio (percentage) of channel pairs that the spatial correlation was significantly lower in pMDD than in control group (rank-sum test, FDR-corrected *p* < 0.05).

Next, we compared spatial correlations between the control group and each of the experimental groups in three oscillations in both eyes-open and eyes-closed conditions. An example of the disruption of spatial correlation in patients with cMDD is shown in Figure 4. For the infraslow amplitude fluctuations in cMDD patients compared to that in the control group, there was prominent disruption of spatial correlation between eyes-closed participants’ theta oscillations in the left frontal – left temporal region (6/27 channel pairs, 22.22%, rank-sum test, FDR-corrected *p* < 0.05) (Figure 5). The most significant channel pair was FC1 - FT7 (rand-sum test, *p* = 0.0043). In pMDD patients relative to the control group, there was prominent disruption of spatial correlations between eyes-open participants’ alpha oscillations in the left occipital – right occipital region (13 / 16 channel pairs, 81.25%, rank-sum test, FDR-corrected *p* < 0.05) (Figure 6). The most significant channel pair was O1 – PO6 (rand-sum test, *p* = 0.0019). For slow amplitude fluctuations, see Supplementary Figures S1, S2.

### 3.3. Breakdown of long-range spatial correlation of theta oscillations of eyes-closed patients with current major depressive disorder

**Figure 7:**
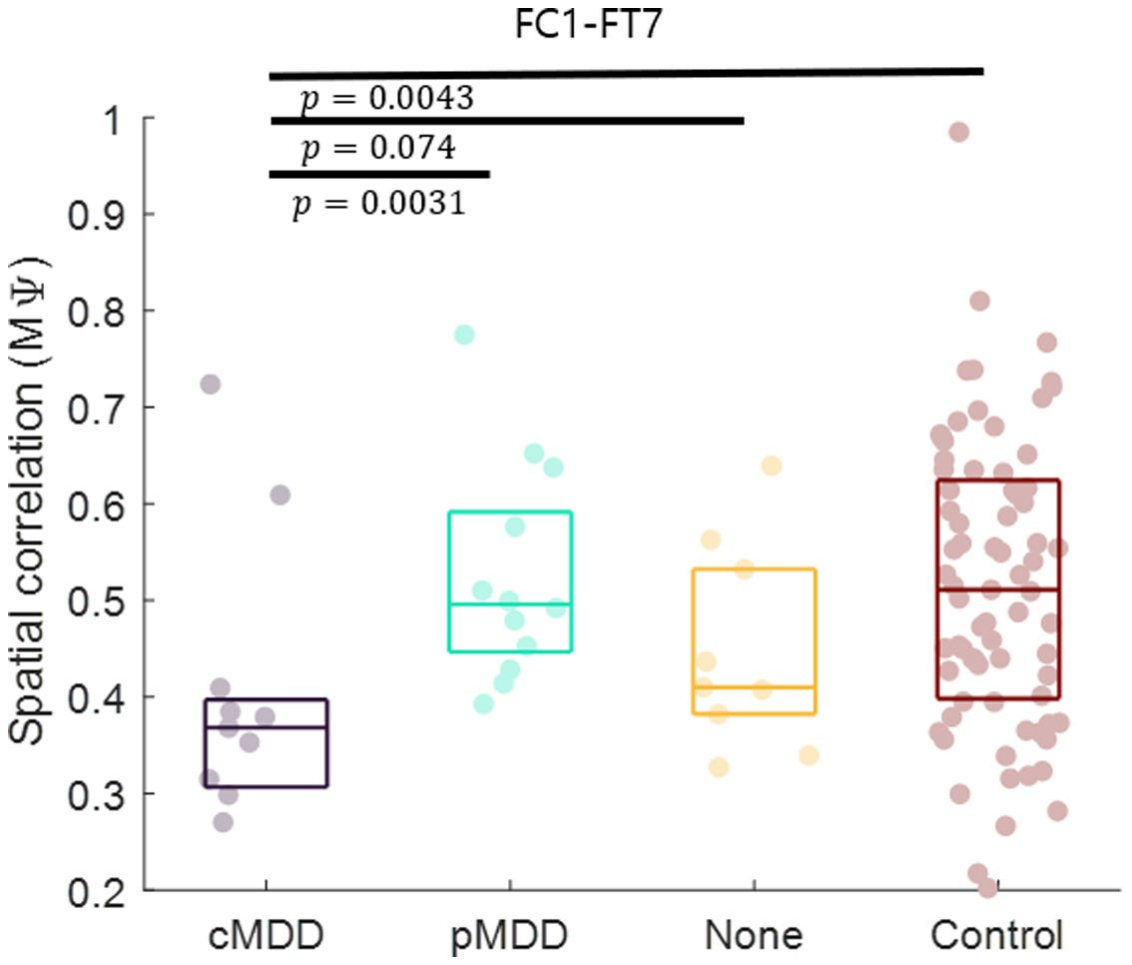
Biomarker as the breakdown of inter-hemispheric long-range spatial correlation between left frontal and temporal region of patients with current major depressive disorder. The mean of spatial correlations ( Mψ in Eq. 2) between channels of left frontal and temporal regions. Each dot represents each participant. The three horizontal lines in the box indicate 25, 50, and 75% of the data, respectively. The result of statistical test that the spatial correlation of the left group was lower than the spatial correlation of the right group was displayed as a *p*-value (rank-sum test).

Since the most statistically significant disruption of spatial correlation occurred in the FC1 – FT7 channels between theta oscillations of eyes-closed patients for current MDD, we set this spatial correlation as the EEG biomarker of current MDD. The spatial correlation between the FC1 and FT7 channels was significantly lower in the cMDD group than in the pMDD group, the None group, or the control group (rank-sum test, *p* = 0.0031, 0.074, and 0.0043, respectively) (Figure 7).

### 3.4. Breakdown of long-range spatial correlation of alpha oscillations of eyes-open patients with past major depressive disorder

**Figure 8:**
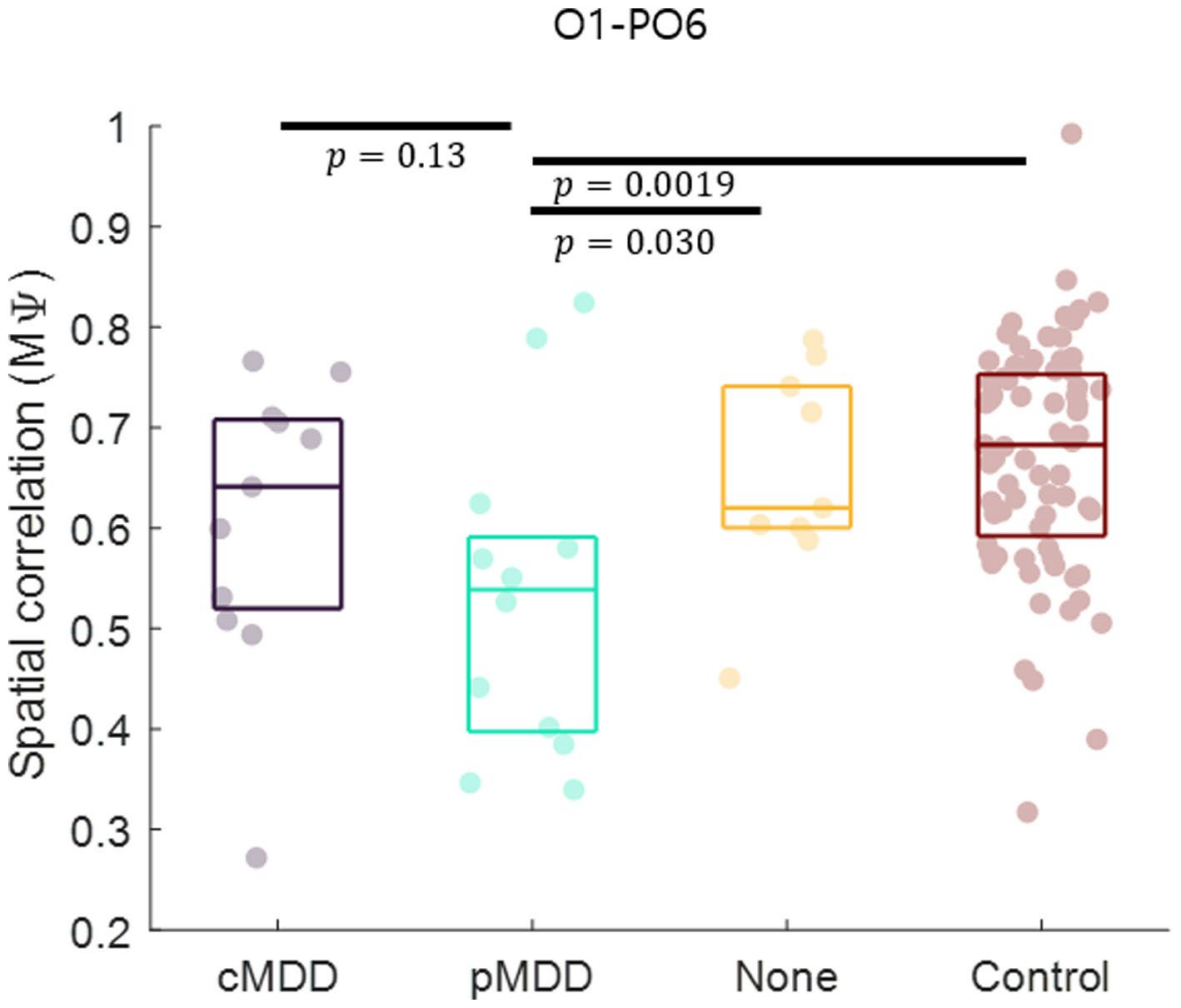
Biomarker as the disruption of inter-regional long-range spatial correlation between left occipital and right occipital region of patients with past major depressive disorder. The mean of spatial correlations ( Mψ in Eq. 2) between channels of left occipital and right occipital regions. Each dot represents each participant. The three horizontal lines in the box indicate 25, 50, and 75% of the data, respectively. The result of statistical test that the spatial correlation in the pMDD group was lower than that in other groups was displayed as a *p*-value (rank-sum test).

Since the most statistically significant breakdown of spatial correlation of past MDD patients occurred in the O1 – PO6 channels for alpha oscillations of eyes-closed patients for past MDD, we set this spatial correlation as the EEG biomarker of past MDD. The spatial correlation between O1 and PO6 channels was significantly lower in the pMDD group than those in the cMDD group, the None group, or the control group (rank-sum test, *p* = 0.13, 0.030, and 0.0019, respectively) (Figure 8).

### 3.5. Discrimination of individuals using biomarkers

**Figure 9:**
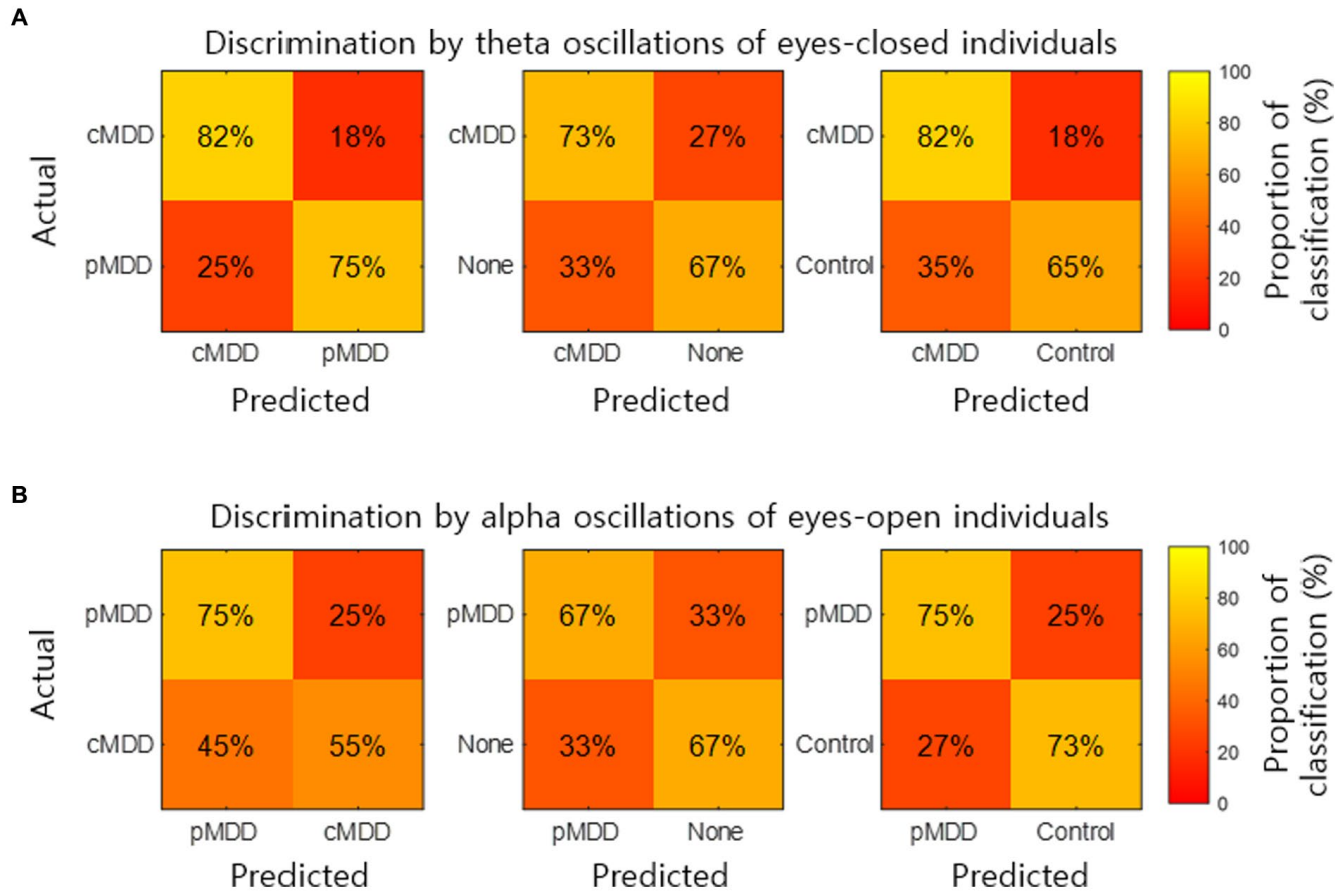
Discrimination of individuals by two kinds of biomarkers. **(A)** The normalized confusion matrix for the 2-classes discrimination (cMDD vs. pMDD, cMDD vs. None, and cMDD vs. Control) using spatial correlations ( Mψ in Eq. 2). **(B)** The normalized confusion matrix for the 2-classes discrimination (pMDD vs. cMDD, pMDD vs. None, and pMDD vs. Control) using spatial correlations ( Mψ in Eq. 2).

The classification of an individual into the cMDD or other groups using spatial correlations of theta oscillations in the eyes-closed condition was evaluated. The accuracy (total classification accuracy), sensitivity (Proportion of individuals of the actual target group discriminated as the target group), and specificity (Proportion of individuals of the actual non-target group discriminated as the non-target group) rates were measured. When discriminating from control groups, cMDD groups had accuracy, sensitivity, and specificity rates of 67, 82, and 65%, respectively, while that for None groups were 70, 73, and 67%. Discrimination between cMDD and pMDD groups had respective rates of 78, 82, and 75% (Figure 9A).

We also evaluate the discrimination of an individual into the pMDD or other groups using spatial correlations between alpha oscillations of eyes-open individuals. Discrimination between the pMDD and control groups had accuracy, sensitivity, and specificity rates of 74, 75, and 73%, respectively, while discriminating between pMDD and None groups had respective rates of 67, 67, and 67%. Discrimination between the pMDD and cMDD groups had rates of 65, 75, and 55% (Figure 9B) with the same respectivity as above.

## 4. Discussion

Many studies have reported that the amplitude fluctuations of EEG oscillations are temporally as well as spatially correlated (10–13). In patients with depression, long-range temporal correlations are reportedly disrupted (15), indicating that the amplitude fluctuations become closer to random (38). In the present study, we hypothesized that if the amplitude fluctuations were close to a random process, spatial correlations of the amplitude fluctuations would also be influenced. We found that the spatial correlation between the amplitude fluctuations of theta oscillations during rest with eyes closed was decreased in individuals with depression. This disruption of spatial correlations was prominent in the left fronto – temporal network for theta oscillations during eye-closed rest, specifically in patients with current MDD (Figure 7). Hence, we propose that spatial correlations of the amplitude fluctuation of EEG theta oscillations between the left frontal and temporal regions can be a potential biomarker for current MDD. Moreover, since the disruption of spatial correlations was prominent in the left occipital – right occipital network for alpha oscillations during eye-open rest, specifically in patients with past MDD, we propose that spatial correlations of the amplitude fluctuation of EEG alpha oscillations between the left occipital and right occipital regions can be a potential biomarker for past MDD.

Along with a previous report about the disruption of long-range temporal correlations (15), the present study demonstrated the disruption of the spatial correlation between theta oscillations. This finding indicates that the amplitude fluctuations of theta oscillations become spatiotemporally close to random in patients with depression. Even so, it is not clear yet whether these decreases in spatial and temporal correlations share the same cause.

**Figure 10:**
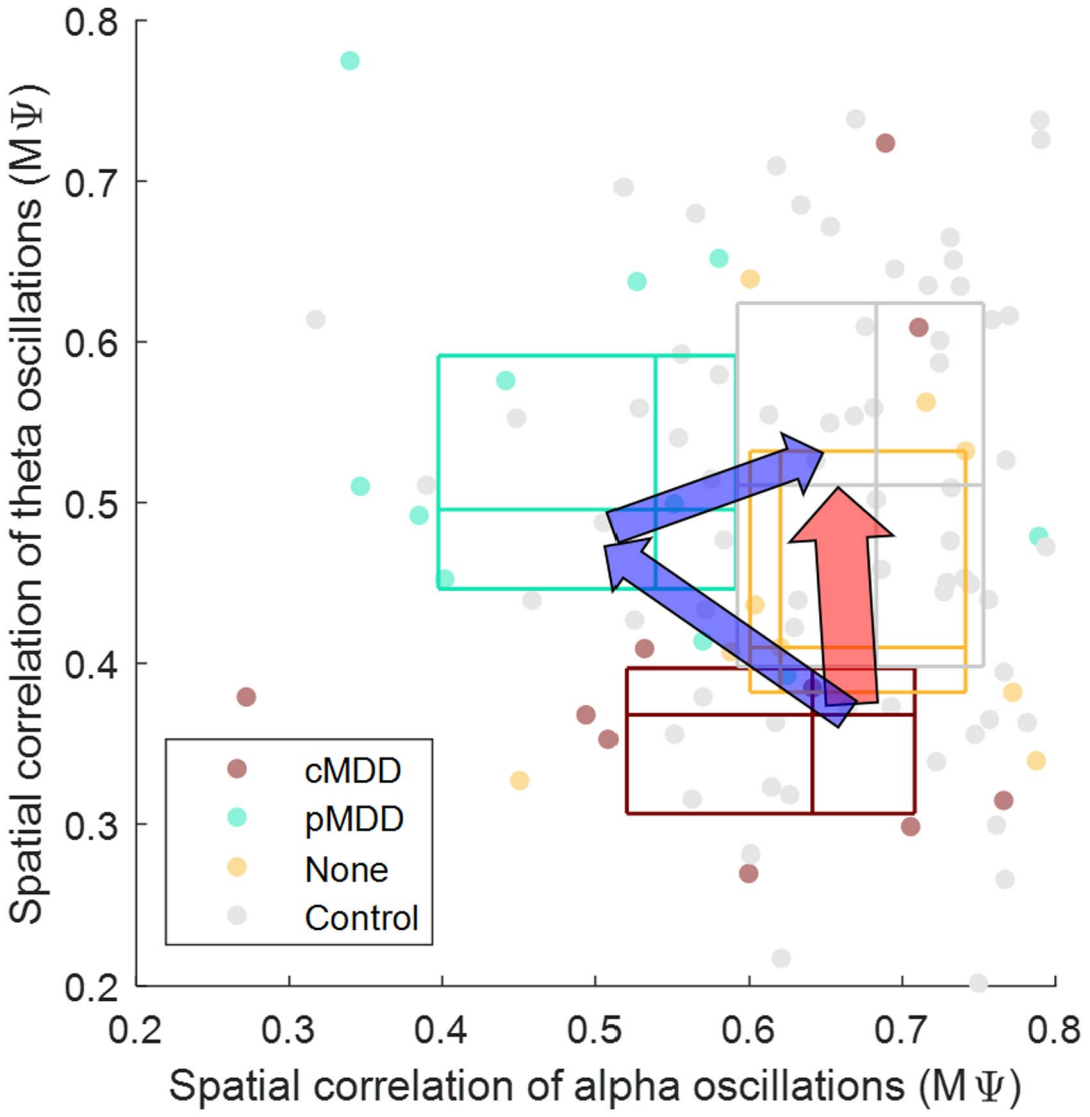
Conceptual representation of different pathways recovering major depressive disorder through biomarkers. The *y*-axis is from Figure 7 ( Mψ in Eq. 2), indicating that the spatial correlation of theta oscillations of eyes-closed individuals. The *x*-axis is from Figure 8 ( Mψ in Eq. 2), indicating that the spatial correlation of alpha oscillations of eyes-open individuals. The boundary lines of each box indicate 25 and 75% of data, respectively. The cross line of each box indicates 50% of data. The red arrow represents the recovery without residual depressive symptoms. The blue arrow represents the recovery with residual depressive symptoms.

Patients with current MDD and past MDD exhibit different patterns of spatial correlation disruptions: theta oscillations under the eyes-closed condition (Figure 5) and alpha oscillations under the eyes-open condition (Figure 8), respectively. This suggests that past MDD patients with depressive symptoms may not simply exist between patients with current MDD and healthy individuals. Since patients with current MDD and individuals with past MDD with depressive symptoms have different EEG biomarkers, this suggests that if recovering individuals have depressive symptoms, they may be in a completely different brain state from individuals who recover without residual depressive symptoms. These biomarkers may help to track the recovery from MDD, as we putatively visualize such tracking on the space composed of spatial correlations of theta and alpha oscillations described above in Figure 10.

The spatial correlation of EEG amplitude fluctuations evaluated in this study measures functional connectivity in the brain. Functional connectivity has been one of the key biomarkers of depression that are found in brain signals (39–41). In previous amplitude-based functional connectivity studies based on rapid transition process (42) or coherence (43), alpha band connectivity was found to increase in patients with depression, which is contrary to our results. The functional connectivity methods used in those previous studies, however, do not focus on gradual changes in amplitude levels thus are different from our evaluation of spatial correlation between amplitude fluctuations. Another study using methods similar to ours, focusing on amplitude envelopes, showed an increase in theta band connectivity in patients with depression (18), which is also contrary to our results. However, we limited amplitude fluctuations to the infraslow frequency band where spatial correlations exist while the other study did not (11–13). Furthermore, functional connectivity is based on the amplitude envelope correlation in the other study, but our study focused on the phase difference of amplitude envelopes instead. It is noteworthy that functional connectivity using amplitude envelope correlations and phase differences have been shown to lead to different results (44). In our study, we included patients with current as well as past MDD. In the case of these past MDD patients, it was reported that the functional connectivity in the beta band increases (24). In our results, however, the functional connectivity in the theta and alpha band in remitted depressed patients was decreased (Figure 8). The functional connectivity studies including the spatial correlation of amplitude fluctuations are vulnerable to EEG volume conduction (45, 46). The effect of EEG volume conduction is considered to be weaker in proportion to the distance (45), and our results mainly drew on long-distance correlations. Therefore, it can be said that the effect of EEG volume conduction in the present study was relatively weak. However, since the effect of EEG volume conduction is not fundamentally eliminated, caution is needed in interpreting the results.

It has been suggested that the amplitude fluctuations of EEG oscillations are correlated with fluctuations in blood-oxygen-level-dependent (BOLD) responses, as shown in a functional magnetic resonance imaging study (47). This may indicate that the spatial correlation of amplitude fluctuations of EEG oscillations could be related to the functional connectivity of BOLD responses between brain regions. From this perspective, a disruption of long-range spatial correlation in patients with depression may be analog to an impairment of long-range functional connectivity of BOLD responses.

The disruption of long-range temporal correlation in relation to EEG oscillations has been reported to be observed not only in patients with depression but also in patients with other mental disorders such as early-stage Alzheimer disease (48) and schizophrenia (49). Therefore, there is a possibility that the disruption of spatial correlations may also appear in patients with other mental disorders apart from depression.

It is challenging to acquire low-frequency range of EEG because a DC-coupled or full-band EEG amplifier is generally required to extract infraslow activity directly from the recorded EEG signals. In contrast, the infraslow amplitude fluctuations investigated in this study are relatively easy to extract because they can be obtained through the filtering and Hilbert transformation of the relatively fast oscillations of EEG (>1 Hz). Consequently, it does not require a DC-coupled EEG amplifier and can therefore be examined more reliably.

This study was limited in that it only involved a restricted use of the infraslow frequency of amplitude fluctuations in relation to EEG oscillations. Due to the short data length, only 0.05–0.1 Hz could be used, excluding slower frequencies (e.g., 0.01–0.1 Hz). Since spatial correlation is stronger in the slower frequency domain (11–13), it is recommended that the disruption of spatial correlation at slower frequencies is investigated in future studies. A second limitation of the present study was the relatively small population size of the patients with depression, preventing a more rigorous validation of the results. One of the limitations of this study is relatively low accuracy of discriminating MDD (65–78%). Some EEG biomarker studies for MDD have reported accuracy data over 90% (19, 20, 41). A reason for the low accuracy obtained in our study would be a high spatial similarity of the infraslow amplitude fluctuations between EEG channels (11–13); this can make discrimination of MDD by spatial correlations difficult. Another possible reason would be that we could use the information in a limited infraslow frequency band (0.05–0.1 Hz) only, due to the limited length of the dataset. Accuracy may improve if we can use information from a wider frequency range (e.g., 0.01–0.1 Hz).

We agree that relatively low discrimination accuracy using infraslow amplitude fluctuations may hinder using this biomarker alone for MDD diagnosis. However, discovering a new biomarker such as the breakdown of spatial correlations of infraslow amplitude fluctuations can be used simultaneously with several existing EEG biomarkers to increase diagnosis accuracy. It has been reported that simultaneous use of multiple biomarkers can increase the accuracy of MDD diagnosis (19, 41). Hence, we believe that the addition of a new EEG biomarker can be beneficial for the development of MDD diagnosis methods.

Many EEG biomarkers for diagnosing MDD have been proposed. These EEG biomarkers include the asymmetry of alpha oscillations, the spectral power of fast oscillations (>1 Hz), evoked potentials, EEG coherence between spatial regions, and phase synchronization, to name a few (see (1)). Although these biomarkers have been used in MDD research, it is useful to find a new biomarker to increase the accuracy of MDD diagnosis (19, 41). Long-range temporal correlation based on the amplitude envelope of EEG has been suggested as a biomarker (15), however it is not known whether the spatial correlations of infraslow amplitude envelopes can provide a new biomarker, which was dealt with in this study.

It is known that frontal alpha asymmetry, one of the biomarkers of depression, can help not only in the diagnosis but also in the treatment of depression. As noted, neurofeedback training for frontal alpha asymmetry has been found to be effective in treating depression (6, 7) while not affecting mood in healthy participants (8). Similar to frontal alpha asymmetry, long-range spatial correlation can be considered as an index of neurofeedback training whereby participants can receive neurofeedback to maintain high long-range spatial correlation. If such neurofeedback treatment is effective, long-range spatial correlation could become an effective biomarker not only in the diagnosis but also in the treatment of depression.

## Data availability statement

The original contributions presented in the study are included in the article/Supplementary material, further inquiries can be directed to the corresponding authors.

## Ethics statement

Ethical review and approval was not required for the study on human participants in accordance with the local legislation and institutional requirements. Written informed consent for participation was not required for this study in accordance with the national legislation and the institutional requirements.

## Author contributions

DS: conceptualization, methodology, software, validation, formal analysis, investigation, resources, data curation, writing – original draft, writing – review and editing, visualization, and project administration. JK: writing – review and editing, supervision, and funding acquisition. O-SK: writing – review and editing, supervision, and project administration. S-PK: writing – original draft, writing – review and editing, visualization, supervision, project administration, and funding acquisition. All authors contributed to the article and approved the submitted version.

## Funding

This research was supported by the Alchemist Brain to X (B2X) Project funded by the Ministry of Trade, Industry and Energy (No. 20012355; NTIS No. 1415181023), the National Research Foundation of Korea (NRF) grant funded by the Korea government (MSIT) (No. RS-2023-00213187), and the U-K Brand Research Fund (1.210046.01) of UNIST (Ulsan National Institute of Science and Technology).

## Conflict of interest

The authors declare that the research was conducted in the absence of any commercial or financial relationships that could be construed as a potential conflict of interest.

## Publisher’s note

All claims expressed in this article are solely those of the authors and do not necessarily represent those of their affiliated organizations, or those of the publisher, the editors and the reviewers. Any product that may be evaluated in this article, or claim that may be made by its manufacturer, is not guaranteed or endorsed by the publisher.
