# Supplementary material for: Breakdown of long-range spatial correlations of infraslow amplitude fluctuations of EEG oscillations in patients with current and past major depressive disorder
Source: Front Psychiatry. 2023 Apr 26;14:1132996. doi: 10.3389/fpsyt.2023.1132996 (PMC10169687; doi:10.3389/fpsyt.2023.1132996)
Supplement: Supplementary file 1 [file Image_1.PDF]

Supplementary Material

Breakdown of long-range spatial correlations of infraslow amplitude fluctuations of EEG oscillations in patients with current and past major depressive disorder

Duho Sihn[1], Ji Sun Kim[2], Oh-Sang Kwon[1*], Sung-Phil Kim[1*]

[1]Department of Biomedical Engineering, Ulsan National Institute of Science and Technology, Ulsan, 44919, Republic of Korea

[2]Department of Psychiatry, College of Medicine, Soonchunhyang University Cheonan Hospital, Cheonan, 31151, Republic of Korea

*** Correspondence:**
Sung-Phil Kim
spkim@unist.ac.kr

Oh-Sang Kwon
oskwon@unist.ac.kr

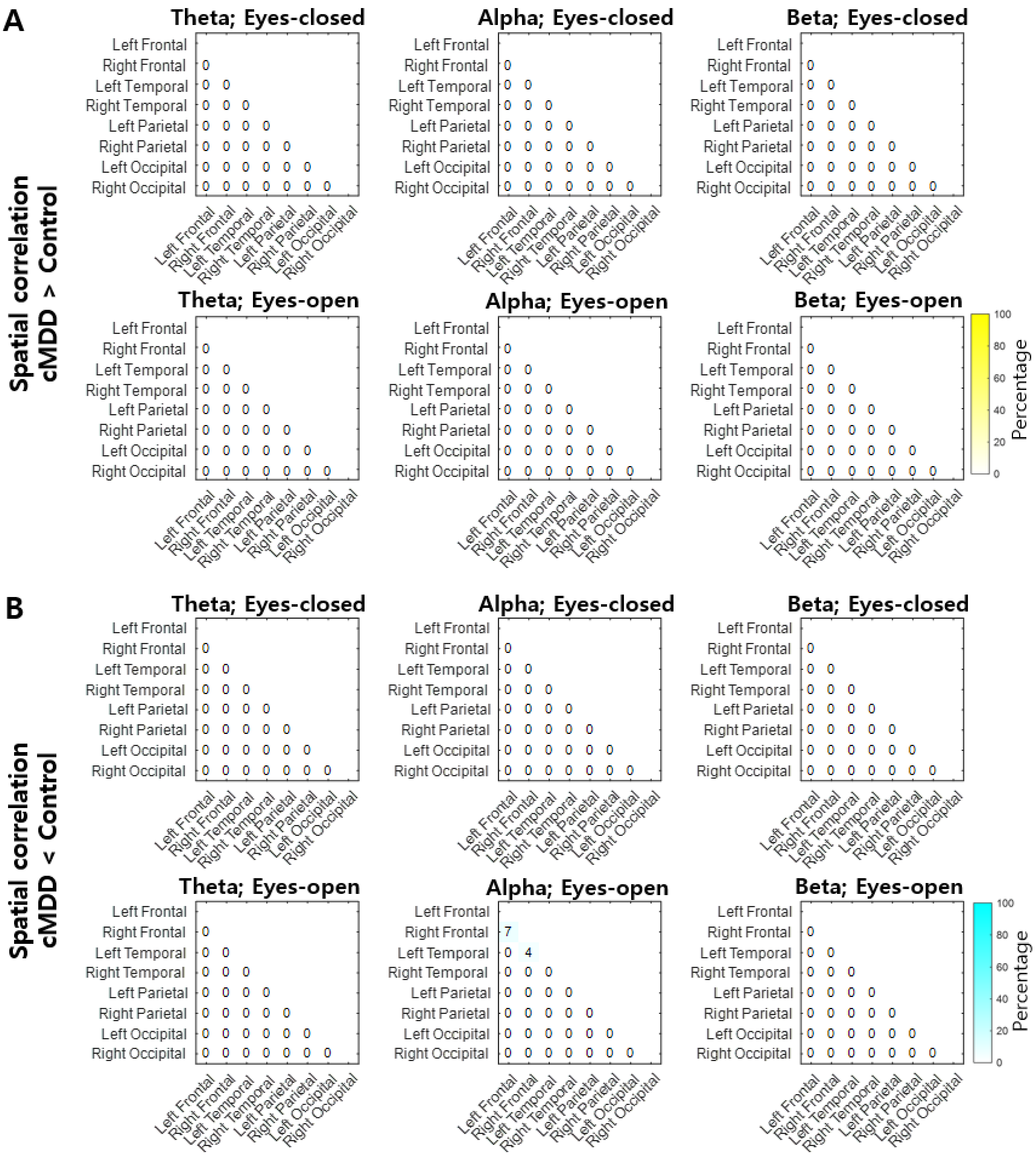

Supplementary Figure 1. Differences of spatial correlation of slow amplitude fluctuations between current major depressive disorder and control groups. (A) The ratio (percentage) of channel pairs that the spatial correlation was significantly higher in cMDD than in control group (rank-sum test, FDR-corrected $p < 0.05$). (B) The ratio (percentage) of channel pairs that the spatial

correlation was significantly lower in cMDD than in control group (rank-sum test, FDR-corrected $p < 0.05$).

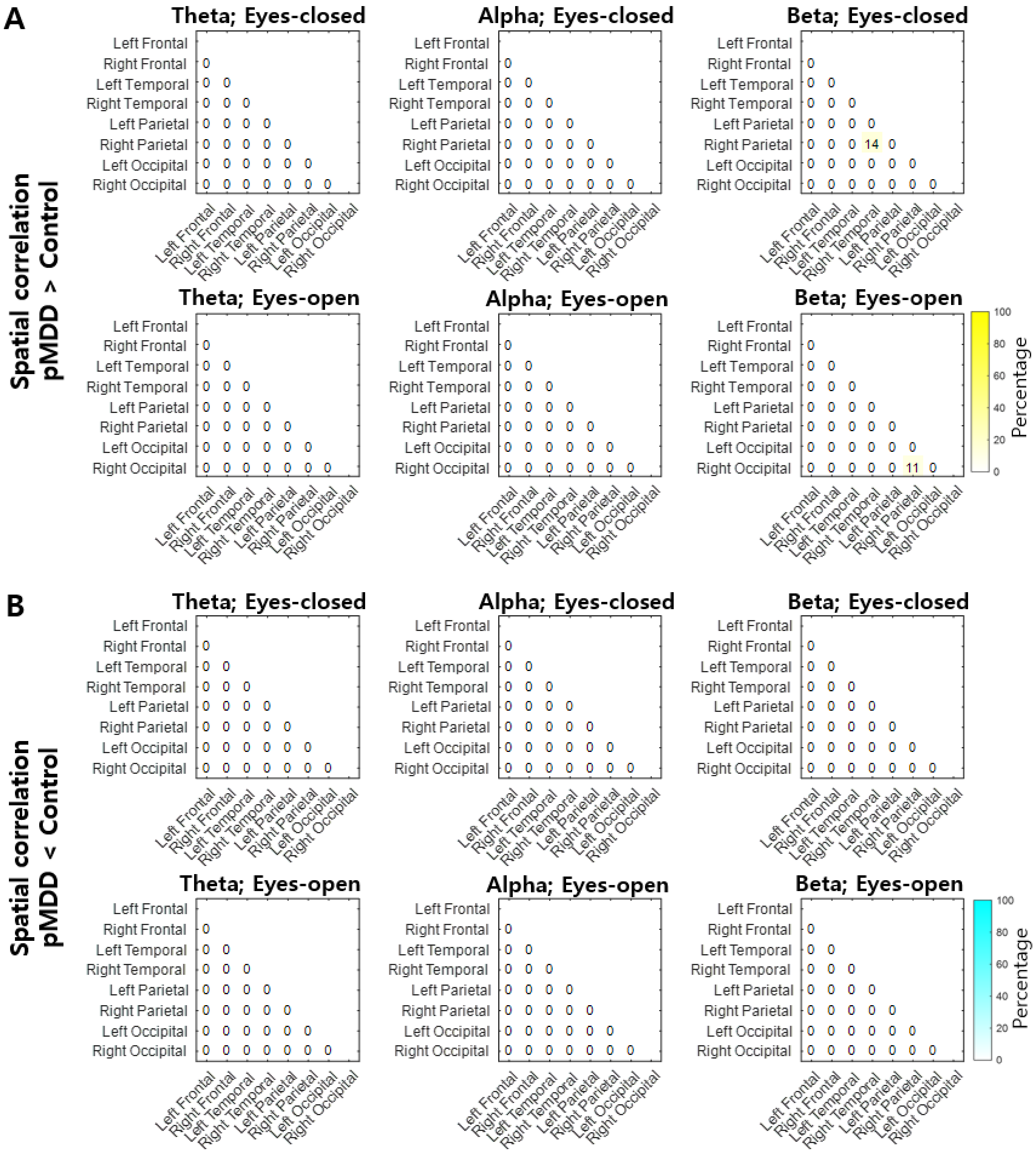

Supplementary Figure 2. Differences of spatial correlation of slow amplitude fluctuations between past major depressive disorder and control groups. (A) The ratio (percentage) of channel pairs that the spatial correlation was significantly higher in pMDD than in control group

(rank-sum test, FDR-corrected $p < 0.05$). (B) The ratio (percentage) of channel pairs that the spatial correlation was significantly lower in pMDD than in control group (rank-sum test, FDR-corrected $p < 0.05$).
